# Incremental Prognostic Value of the CONUT Score for In-Hospital Mortality and Length of Stay in Hospitalized Patients

**DOI:** 10.3390/nu18081249

**Published:** 2026-04-15

**Authors:** Jose M. Romero-Márquez, Cristina Novo-Rodríguez, María Hayón-Ponce, María Novo-Rodríguez, Araceli Muñoz-Garach, Victoria Luna-López, Carmen Tenorio-Jiménez

**Affiliations:** 1 Department of Endocrinology and Nutrition, Virgen de las Nieves University Hospital, 18014 Granada, Spain; romeromarquez@ugr.es (J.M.R.-M.); cristinanovorodriguez@gmail.com (C.N.-R.); mahayon21@gmail.com (M.H.-P.); marianovor@hotmail.com (M.N.-R.); aracelimugar@gmail.com (A.M.-G.); vlunal@yahoo.es (V.L.-L.); 2 Foundation for Biosanitary Research of Eastern Andalusia—Alejandro Otero (FIBAO), 18012 Granada, Spain; 3 Department of Nutrition and Sports Sciences, Faculty of Health Sciences, International University of La Rioja, 26006 La Rioja, Spain; 4 Granada Biosanitary Research Institute (ibs.GRANADA), 18012 Granada, Spain; 5 Physiopathology of Obesity and Nutrition Networking Biomedical Research Centre (CIBEROBN), Carlos III Health Institute, 28029 Madrid, Spain; 6 Department of Medicine, Faculty of Medicine, University of Granada, 18016 Granada, Spain

**Keywords:** prognostic biomarkers, immunometabolic marker, CONUT score, in-hospital mortality, length of stay, hospital outcomes, risk stratification

## Abstract

Background: Disease-related malnutrition is highly prevalent among hospitalized patients and is associated with increased mortality, complications, and prolonged hospital stays. Early identification of patients at nutritional risk is therefore essential to improve clinical outcomes. The Controlling Nutritional Status (CONUT) score is an objective prognostic immunometabolic marker derived from serum albumin, total cholesterol, and lymphocyte count. This study aimed to evaluate the prognostic value of the CONUT score for in-hospital mortality and length of hospital stay (LOS) in hospitalized patients with moderate-to-severe nutritional risk and to determine whether incorporating CONUT improves the predictive performance of a clinical model based on routine admission variables. Methods: A retrospective observational cohort study was conducted, including 671 adult patients admitted to a tertiary university hospital with CONUT ≥ 6. Multivariable logistic regression was used to assess predictors of in-hospital mortality, while LOS was analyzed using multivariable linear regression. Model discrimination was evaluated using receiver operating characteristic (ROC) curve analysis and comparison of the area under the curve (AUC). Results: Higher CONUT scores were independently associated with increased in-hospital mortality. Each one-point increase in CONUT was associated with 28% higher odds of death (OR 1.28; 95% CI 1.13–1.46; *p* < 0.001). Patients with a severe CONUT score had significantly higher mortality compared with those with a moderate CONUT score (OR 1.77; 95% CI 1.12–2.81; *p* = 0.004). Incorporating CONUT into the clinical prediction model significantly improved discrimination, increasing the AUC from 0.728 to 0.753 (DeLong *p* = 0.035). Higher CONUT values were also associated with longer hospital stays: each additional point corresponded to a 5.4% increase in LOS (*p* = 0.009), and a severe CONUT score was associated with a 17.6% longer stay (*p* = 0.027). Conclusions: the CONUT score is independently associated with in-hospital mortality and prolonged hospitalization. While its incremental discriminative improvement is modest, its automated calculation from routine laboratory data makes it a practical and scalable tool for early risk stratification.

## 1. Introduction

Disease-related malnutrition is highly prevalent among hospitalized patients, with approximately 20–50% presenting nutritional risk or overt malnutrition at admission [1]. A substantial body of evidence has shown that poor nutritional status at hospital entry is associated with adverse outcomes, including increased mortality, higher complication rates, prolonged hospital stays, and greater healthcare costs [2,3]. For this reason, early identification of nutritional risk has become a priority in hospital medicine. Current recommendations from the European Society for Clinical Nutrition and Metabolism and the Global Leadership Initiative on Malnutrition (GLIM) emphasize the importance of systematic nutritional screening at hospital admission and the use of standardized diagnostic processes, recognizing baseline nutritional status as an important determinant of hospital prognosis [4,5].

In routine clinical practice, several screening tools are commonly used to identify patients at nutritional risk, including the Nutritional Risk Screening 2002 (NRS-2002), the Malnutrition Universal Screening Tool (MUST), and the Subjective Global Assessment (SGA) [6,7,8]. Although these instruments are well validated, their systematic implementation in busy hospital environments remains challenging. They often require patient interviews, physical examination, and anthropometric measurements such as weight and height, information that is not always readily available at the time of admission. Moreover, part of the evaluation relies on subjective clinical judgment, which may introduce variability across observers. These practical limitations have stimulated interest in automated approaches to risk stratification based on routinely available laboratory parameters.

The CONUT score was developed as an automated hospital risk assessment and surveillance system, designed to stratify patients according to immunometabolic and disease-related risk in hospitalized patients using routinely collected biochemical data [9]. The score integrates three laboratory parameters (serum albumin, total cholesterol, and lymphocyte count), which reflect not only nutrition-related parameters but also systemic inflammation, disease burden, and metabolic stress. Because these variables are typically measured as part of routine admission blood tests, CONUT provides an objective, reproducible, and low-cost index of prognostic factors that can be automatically calculated and integrated into electronic health record systems without increasing clinical workload [9].

An increasing number of studies suggest that the CONUT score also carries important prognostic information across different inpatient settings. In a prospective cohort of patients admitted to Internal Medicine and Gastroenterology departments, higher CONUT scores at admission were associated with both increased in-hospital mortality and longer hospital stays [10]. Similar findings have been reported in internal medicine cohorts, where higher CONUT values were linked to mortality, sepsis, and prolonged hospitalization [11]. Among hospitalized older adults, CONUT has likewise been associated with mortality, complications, and extended length of stay [12]. Comparable associations have also been described in specific clinical contexts, including acute heart failure [13], acute ischemic stroke [14], and patients undergoing gastrectomy for gastric cancer [15], suggesting that the score may capture a broader phenotype of physiological vulnerability across diverse disease states.

Despite these findings, several important gaps remain. Existing studies show substantial variability in population characteristics, clinical settings, and the cut-off values used to define CONUT risk strata. In addition, CONUT has been analyzed inconsistently as either a categorical or continuous variable, limiting comparability across studies and hindering a clear understanding of its optimal use as a prognostic indicator [10,12]. Furthermore, relatively few investigations have simultaneously evaluated both in-hospital mortality and length of hospital stay, and the incremental predictive value of CONUT beyond routinely available clinical variables at admission remains insufficiently explored [11]. Addressing these issues is particularly relevant in real-world hospital populations, where the evaluation of CONUT using both analytical approaches may improve its interpretability and facilitate scalable, automated risk stratification without increasing clinical workload.

Therefore, the aim of the present study was to evaluate the prognostic value of the CONUT score for in-hospital mortality and length of hospital stay in a real-world cohort of hospitalized adults with moderate-to-severe CONUT scores. In addition, we assessed whether incorporating CONUT into a clinical prediction model based on routinely available admission variables improves its discriminative performance.

## 2. Materials and Methods

### 2.1. Study Design, Patients and Ethical Approval

**Figure 1:**
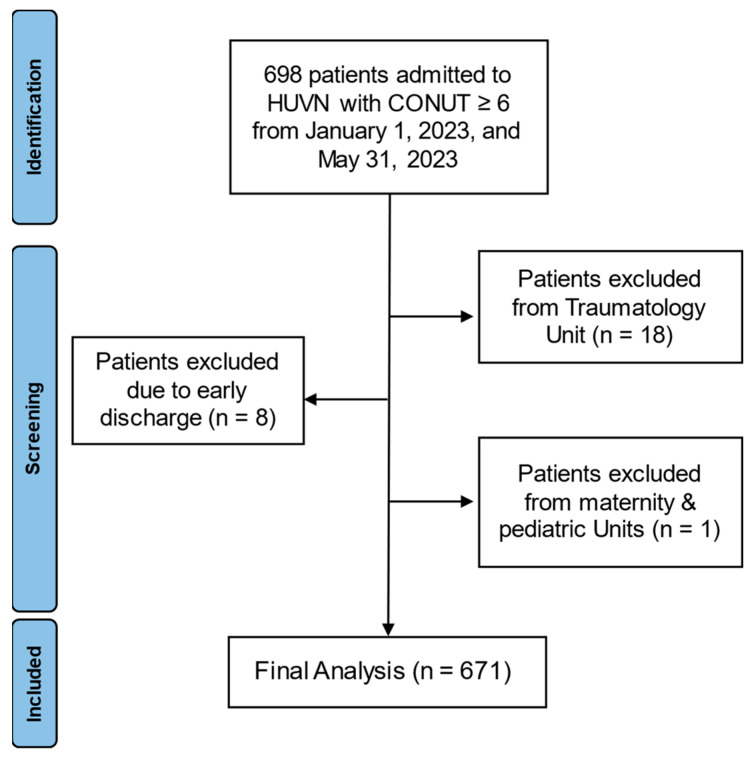
Study flow diagram: patient identification, screening, and inclusion process.

A single-center, retrospective observational cohort study was conducted. All adults aged ≥18 years admitted to any service of Hospital Universitario Virgen de las Nieves (Granada) between 1 January 2023 and 31 May 2023 were considered for inclusion. Patients were included if they had a complete blood count available, acknowledging that laboratory testing was performed at the discretion of the attending physician. Patients with early discharge (<24 h) or in terminal condition were excluded, as well as those admitted to intensive care, trauma, and maternity/pediatric units (Figure 1).

### 2.2. Data Collection and Variables

Clinical and demographic data were obtained through a systematic review of the hospital’s electronic medical records. The study included all adult patients admitted during the study period who presented a positive alert in the CONUT nutritional screening system. In the study hospital, the CONUT score is automatically calculated from routine laboratory parameters (serum albumin [g/dL], total lymphocyte count [cells/mm^3^], and total cholesterol [mg/dL]) and integrated into the laboratory information system. By default, CONUT values are only exported automatically to the patient’s electronic health record when the score is ≥6, corresponding to at least moderate nutritional risk. Consequently, patients with a CONUT score of 5, although classified as moderate risk according to standard criteria, were not identifiable in the electronic records and could not be included in the analysis. In line with the real-world design of the study, this approach was intentionally maintained to reflect routine clinical workflows and avoid introducing artificial recalculations or potential selection bias. Patients were subsequently categorized into moderate and severe nutritional risk groups according to the standard CONUT classification.

For each patient, demographic information, including age and sex, was collected, together with data related to hospital admission characteristics. These included the type of admission (medical or surgical), referral to the nutrition unit during hospitalization, and the administration of nutritional support. The primary diagnosis at admission was extracted from the clinical records and categorized according to the International Classification of Diseases, Tenth Revision (ICD-10).

Additionally, the hospital department responsible for the patient’s admission was recorded, including Internal Medicine, Gastroenterology, General Surgery, Pulmonology, Cardiology, Medical Oncology, Infectious Diseases, Nephrology, Hematology, Urology, Angiology and Vascular Surgery, and other medical or surgical specialties. Finally, clinical outcomes during hospitalization were collected, specifically length of hospital stay and in-hospital mortality. These variables were used to describe the clinical characteristics and outcomes of hospitalized patients presenting moderate or severe nutritional risk as identified by the CONUT screening system.

### 2.3. Statistical Analysis

All analyses were performed using Jamovi (version 2.3.28). A two-sided *p*-value < 0.05 was considered statistically significant. Continuous variables were described as mean ± standard deviation (SD) or median (interquartile range, IQR), as appropriate, and categorical variables were expressed as frequencies and percentages.

The primary outcome was in-hospital mortality (alive vs. deceased). To evaluate the independent association between nutritional status and mortality, hierarchical multivariable logistic regression models were constructed. Age was included as a continuous covariate, while gender, ICD-10-based diagnostic, admission service, and CONUT malnutrition risk severity were entered as categorical predictors with predefined reference categories (female, respiratory disease, internal medicine service admission, and moderate CONUT Score).

Model development followed a sequential approach to assess the incremental prognostic value of the CONUT score: (1) Model 1 included clinical variables (age, sex, ICD-10-based diagnostic, and admission service); (2) Model 2 consisted of Model 1 plus CONUT as a continuous variable (per-point increase); (3) Model 3 consisted of Model 1 plus CONUT as a categorical variable (severe [CONUT score: 9–12] vs. moderate [CONUT score: 6–8]).

Odds ratios (OR) with 95% confidence intervals (CI) were reported. Model performance was evaluated using likelihood ratio tests, Akaike Information Criterion (AIC), and Nagelkerke’s pseudo-R^2^. Discriminative ability was assessed using ROC curves and the AUC. Differences between AUCs were assessed using DeLong’s test for correlated ROC curves. Sensitivity and specificity were calculated using a probability threshold of 0.5, although model discrimination was primarily interpreted using AUC. Multicollinearity was assessed using variance inflation factors (VIF), with all predictors showing values close to 1 (range 1.02–1.18), indicating the absence of relevant collinearity. For models including CONUT as a continuous variable, the linearity assumption in the logit was evaluated using a Box–Tidwell approach; the interaction term between CONUT and its natural logarithm was not statistically significant (*p* = 0.591), confirming linearity in the logit.

LOS, measured in days, was analyzed as a secondary outcome. Given the right-skewed distribution of LOS, a natural logarithmic transformation was applied prior to modeling to approximate normality and stabilize variance. Log-transformed LOS was used as the dependent variable in multivariable linear regression analyses.

Three complementary models were constructed to assess the association between nutritional status and LOS: Model A included clinical variables (age, sex, ICD-10-based diagnostic, and admission service); Model B consisted of Model 1 plus CONUT as a continuous variable (per-point increase); and Model C consisted of Model 1 plus CONUT as a categorical variable (severe vs. moderate). All models were adjusted for age, sex (reference: female), ICD-10-based diagnostic (reference: respiratory diseases), and admission specialty (reference: internal medicine). Regression coefficients (β) with 95% CI were reported and, for clinical interpretability, results were additionally expressed as percentage change in LOS, calculated as (e^β − 1) × 100. Model assumptions were evaluated through inspection of residual-versus-fitted plots and Q–Q plots of standardized residuals, and influential observations were examined using Cook’s distance. Multicollinearity was reassessed using VIF in these models.

Time-to-discharge was additionally explored using Kaplan–Meier survival analysis. LOS was used as the time variable, and hospital discharge was defined as the event of interest, with in-hospital death treated as a censoring event. Patients were stratified according to CONUT score severity, and differences between curves were evaluated using the log-rank test.

## 3. Results

### 3.1. Baseline Characteristics

A total of 671 hospitalized patients were included in the analysis, of whom 497 (74.1%) presented a moderate CONUT score and 174 (25.9%) a severe CONUT score according to the CONUT score (Table 1). The overall mean age was 74.2 ± 14.4 years, and 40.7% were women. Patients with severe CONUT scores were slightly younger on average (72.0 ± 14.7 years) compared with those with moderate CONUT scores (75.0 ± 14.3 years).

Most admissions corresponded to medical services (80.7%), whereas 19.3% were surgical admissions. Referral to the Nutrition Unit occurred in 15.1% of medical admissions and 5.1% of surgical admissions, and 18.3% of patients received nutritional support during hospitalization.

Regarding underlying diagnoses, the most frequent ICD-10 categories were respiratory diseases (25.5%), digestive diseases (25.0%), and circulatory diseases (15.5%), followed by neoplasms (11.8%) and genitourinary diseases (10.0%). Severe CONUT score was proportionally more frequent among patients admitted with respiratory diseases (6.2% of the total cohort) and digestive diseases (8.0%).

Hospital admission services were predominantly Internal Medicine (38.7%), followed by Gastroenterology (14.7%), General Surgery (11.0%), and Pulmonology (6.8%), with smaller proportions distributed among other medical and surgical specialties.

The median length of hospital stay was 11 days (IQR 12) overall. Patients with severe CONUT score showed a not significant longer hospitalization (median 13 days, IQR 14.3) compared with those with moderate CONUT score (median 11 days, IQR 12.0).

Overall, in-hospital mortality was 19.6% (n = 132). Mortality was notably higher among patients with severe CONUT score (47 deaths) compared with those with moderate CONUT score (85 deaths), suggesting a clinically relevant association between worse nutritional status and adverse hospital outcomes.

### 3.2. CONUT Score as a Mortality Predictor

Results from hierarchical multivariable logistic regression models evaluating predictors of in-hospital mortality are presented in Table 2. Increasing age was independently associated with a higher risk of in-hospital death across all models, with each additional year of age associated with approximately a 2–3% increase in mortality risk (Model 3: OR 1.03, 95% CI 1.01–1.05; *p* < 0.001). Sex was not significantly associated with mortality in any model. Admission service showed a significant overall association with mortality across all models (global *p* < 0.001). At the individual level, Cardiology and General Surgery were independently associated with lower odds of mortality (Table S1), whereas Hematology and Medical Oncology were associated with higher mortality compared with Internal Medicine (Table S1). In contrast, the ICD-10 diagnostic category was not independently associated with mortality.

When CONUT was incorporated as a prognostic indicator into the models, higher CONUT scores were independently associated with increased mortality risk. In the model including CONUT as a continuous variable, each one-point increase in CONUT was associated with 28% higher odds of in-hospital death (Model 2: OR 1.28, 95% CI 1.13–1.46; *p* < 0.001). Similarly, when analyzed categorically, patients with a severe CONUT score showed significantly higher mortality compared with those with a moderate CONUT score (Model 3: OR 1.77, 95% CI 1.12–2.81; *p* = 0.004).

The inclusion of the CONUT score also improved the overall predictive performance of the models. The clinical model (Model 1) demonstrated modest explanatory capacity (Nagelkerke’s R^2^ = 0.177) and moderate discriminative ability with an AUC of 0.728 (95% CI 0.682–0.775) and an AIC of 630. Incorporating CONUT as a continuous variable (Model 2) resulted in the best overall performance, with the lowest AIC (618), the highest explained variance (R^2^ = 0.206), and the strongest discrimination (AUC = 0.753; 95% CI 0.709–0.797). The model including categorical CONUT (Model 3) also improved model performance compared with the clinical model (AIC = 626; R^2^ = 0.189; AUC = 0.742; 95% CI 0.697–0.787), although performance remained slightly lower than that observed with the continuous specification.

**Figure 2:**
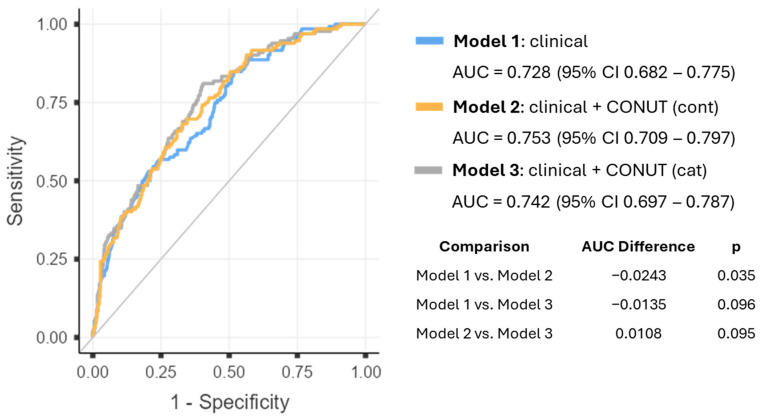
ROC curves comparing three models for the prediction of in-hospital mortality. Model 1 includes clinical variables (age, sex, ICD-10–based diagnosis, and admission service). Model 2 includes Model 1 plus CONUT as a continuous variable (per-point increase). Model 3 includes Model 1 plus CONUT as a categorical variable (severe vs. moderate). The diagonal grey line represents the line of no discrimination (AUC = 0.5), corresponding to random prediction performance. Reference categories are female sex, internal medicine admission, respiratory diseases (ICD-10 chapter), and moderate malnutrition. AUC values are presented with 95% confidence intervals. Statistical comparison was performed using DeLong’s test.

ROC curves comparing the three hierarchical models for the prediction of in-hospital mortality are shown in Figure 2. The clinical model (Model 1), including age, sex, ICD-10 diagnostic chapter, and admission service, demonstrated moderate discriminative ability with an AUC of 0.728 (95% CI 0.682–0.775). The addition of the CONUT score as a continuous variable (Model 2) improved discrimination, reaching the highest AUC of 0.753 (95% CI 0.709–0.797). When CONUT was incorporated as a categorical variable (Model 3), the model also showed improved discrimination compared with the clinical model, with an AUC of 0.742 (95% CI 0.697–0.787), although the performance was slightly lower than that observed with the continuous specification.

Pairwise comparisons using DeLong’s test showed that the improvement in discriminative ability between the clinical model and the model including continuous CONUT was statistically significant (*p* = 0.035). In contrast, the difference between the clinical model and the categorical CONUT model did not reach statistical significance (*p* = 0.096), and no significant difference was observed between the two CONUT models (*p* = 0.095). These findings indicate that incorporating CONUT into the clinical model improves mortality prediction, particularly when the score is analyzed as a continuous variable.

### 3.3. CONUT Score as a Length of Stay Predictor

The association between inmunometabolic risk and LOS was evaluated using multivariable linear regression models with log-transformed LOS as the dependent variable (Table 3). Increasing age was independently associated with shorter hospitalization, with each additional year corresponding to an approximate 0.6% reduction in LOS across all models (Model C: β −0.00559, 95% CI −0.0103 to −0.00084; *p* = 0.021). Sex was not significantly associated with LOS in any model.

Admission service showed a significant overall association with LOS in the clinical model (global *p* = 0.002). At the individual level, Gastroenterology was associated with shorter LOS, whereas Hematology was associated with longer LOS (Table S2). In contrast, the ICD-10 diagnostic category also showed a significant overall association with LOS (global *p* = 0.001), with several categories, particularly circulatory and digestive diseases, as well as other chapters, consistently associated with increased LOS across all models.

When CONUT was incorporated into the models, higher CONUT scores were associated with longer hospitalization. In the model including CONUT as a continuous variable (Model B), each one-point increase in CONUT was associated with a 5.4% increase in LOS (β = 0.05227, 95% CI 0.0130–0.0915; *p* = 0.009). Similarly, when CONUT score severity was analyzed categorically, patients with severe CONUT score had significantly longer hospital stays compared with those with moderate CONUT score, corresponding to a 17.6% increase in LOS (β = 0.16197, 95% CI 0.0187–0.3052; *p* = 0.027).

Overall model fit for the log-linear regression models was statistically significant (global F-test *p* < 0.001). The clinical model explained a modest proportion of LOS variability (R^2^ = 0.0925; adjusted R^2^ = 0.0631). Incorporation of CONUT slightly improved explanatory performance, with R^2^ = 0.102 (adjusted R^2^ = 0.0715) for the continuous specification and R^2^ = 0.099 (adjusted R^2^ = 0.069) for the categorical specification, indicating comparable model performance. No evidence of multicollinearity was observed (VIF range 1.02–1.18).

**Figure 3:**
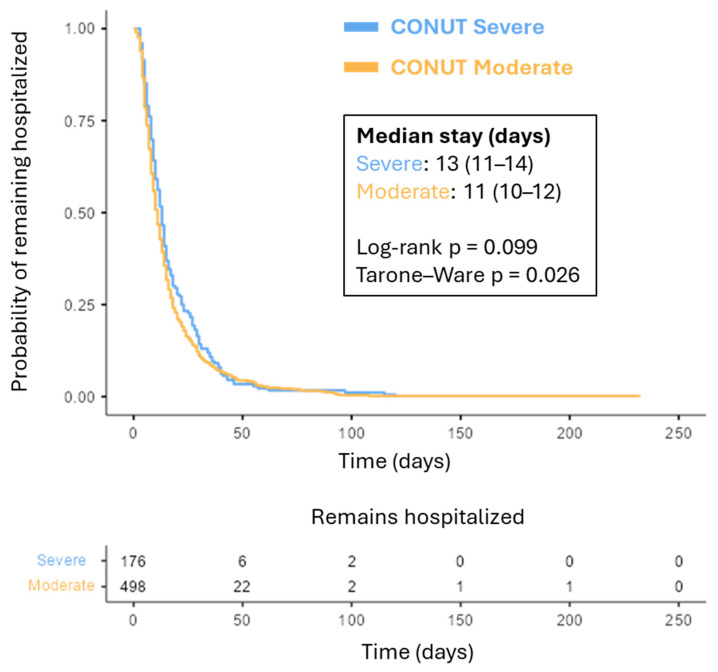
Kaplan–Meier curves for time to discharge according to CONUT severity. Curves are stratified by CONUT severity. Death was treated as a censoring event. Differences were assessed using the log-rank and Tarone–Ware tests.

Kaplan–Meier analysis for time to discharge showed that patients with severe CONUT scores tended to remain hospitalized longer than those with moderate CONUT scores (Figure 3). Median LOS was 13 days (95% CI 11–14) in the severe group compared with 11 days (95% CI 10–12) in the moderate group. Differences between groups were statistically significant using the Tarone–Ware test (*p* = 0.026), while the log-rank test showed a similar trend (*p* = 0.099). Together, these findings indicate that worse nutritional status assessed by CONUT is independently associated with prolonged hospitalization, supporting the clinical relevance of nutritional assessment in predicting hospital resource utilization.

## 4. Discussion

The primary aim of this study was to evaluate the prognostic utility of the CONUT score for predicting in-hospital mortality and LOS in a real-world cohort of hospitalized adults. In this cohort of 671 patients with moderate to elevated baseline risk (CONUT ≥ 6), the score was independently associated with both in-hospital mortality and prolonged hospital stay. Specifically, patients with a severe CONUT score (≥9) exhibited a significantly higher risk of in-hospital mortality (OR 1.77; 95% CI 1.12–2.81; *p* = 0.004) compared to those with a moderate score. Modeled continuously, each one-point increase in CONUT raised the odds of death by 28% (OR 1.28; 95% CI 1.13–1.46; *p* < 0.001) and increased hospital stay by 5.4% (*p* = 0.009). Furthermore, incorporating CONUT into standard clinical models resulted in a modest but significant improvement in discriminative prognostic performance (ROC increased from 0.728 to 0.753; *p* = 0.035). Importantly, this prognostic gradient remained robust even within a cohort already enriched for elevated baseline risk. Beyond mortality risk, the CONUT score was also associated with hospital resource utilization. A clear dose–response relationship was observed between CONUT and LOS: each one-point increase in the score corresponded to a 5.4% increase in hospital stay (*p* = 0.009), and patients with severe CONUT scores experienced hospitalizations that were 17.6% longer than those with moderate CONUT scores (*p* = 0.027).

These findings not only confirm the independent prognostic relevance of CONUT in this high-risk population but are also consistent with a growing body of evidence across heterogeneous hospitalized populations where systemic inflammation and nutritional depletion frequently overlap. The CONUT score was originally developed as an automated nutritional surveillance system designed to facilitate early detection of hospital malnutrition using routine biochemical parameters obtained at admission [9]. However, as noted by recent evidence, its components (serum albumin, total cholesterol, and total lymphocyte count) are not only proxies for nutritional reserves but also reflect systemic inflammation, disease burden, and immune response [16]. Since its introduction, multiple studies have demonstrated that higher CONUT scores are associated with adverse outcomes in hospitalized patients. In cohorts of medical and geriatric inpatients, elevated CONUT has been independently associated with both short- and long-term mortality. For example, in geriatric populations, higher admission CONUT has been linked to increased 30-day and one-year mortality [17], while studies in mixed hospitalized medical populations have reported associations with in-hospital complications and longer length of stay [18,19]. In hospitalized patients, early nutritional support has been associated with reduced in-hospital mortality; however, oral nutritional supplements do not appear to significantly modify CONUT scores, and no clear association has been observed between supplementation and improvements in CONUT classification, particularly in palliative settings [10,20]. In these patients, disease-related inflammation and clinical severity outweigh the potential impact of nutritional interventions, suggesting that CONUT reflects overall disease burden rather than isolated nutritional intake. In this context, the prognostic strength of CONUT may be largely driven by its ability to capture the combined effect of nutritional impairment and systemic inflammatory–metabolic stress associated with underlying disease severity [16,21].

Within this broader evidence framework, our findings show a high degree of consistency with previous studies conducted in comparable clinical settings, particularly those involving internal medicine and gastroenterology populations, which represented a substantial proportion of admissions in our cohort. In a prospective Italian study including patients admitted to internal medicine and gastroenterology wards, moderate-to-severe CONUT Score at admission predicted in-hospital mortality with good discrimination, and the optimal prognostic cut-off was close to the severity threshold used in the present analysis [10]. Similarly, in a broader Spanish hospital cohort, CONUT score was associated with increased mortality, and the score demonstrated superior discriminative performance compared with SGA and NSA in ROC analyses, reinforcing its pragmatic value as an automatable risk assessment tool within real-world hospital workflows [22]. In the present study, the association between CONUT and hospital length of stay also follows the direction reported in previous Spanish and Italian cohorts, despite differences in study design and the clinical and risk profiles included [10,22]. Importantly, although our analysis was restricted to patients with CONUT ≥ 6, thereby reducing score variability and potentially attenuating discriminative capacity, a clear gradient in both mortality and LOS remained evident. This observation suggests that CONUT retains sensitivity to clinically meaningful differences even within populations already enriched for elevated clinical and inflammatory burden.

Collectively, this evidence extends the applicability of CONUT beyond general medical populations, with consistent associations observed in cardiovascular and multimorbidity contexts. In the multicenter NONAVASC registry, CONUT was independently associated with one-year mortality in very elderly patients hospitalized with non-valvular atrial fibrillation, supporting the concept that the score captures a phenotype of reduced physiological reserve and systemic vulnerability extending beyond the acute hospitalization [23]. Similar associations have been described in chronic coronary disease, where higher CONUT scores were independently associated with long-term mortality, with moderate discriminative capacity [24]. At the population level, analyses of cardiovascular disease cohorts derived from national surveys have shown that higher CONUT scores are associated with both all-cause and cardiovascular mortality, with ROC performance comparable or superior to BMI when demographic factors are considered [25]. Additionally, studies in structural cardiac procedures have reported associations between preoperative CONUT and adverse outcomes, suggesting that this index may have relevance for peri-procedural risk stratification based on global clinical status [26,27].

From a mechanistic perspective, these consistent associations across diverse clinical contexts are supported by the biological plausibility of CONUT as a composite marker of immuno-metabolic vulnerability. This plausibility derives from the pathophysiological information captured by its three components (serum albumin, total cholesterol, and total lymphocyte count), each of which reflects partially overlapping mechanisms associated with adverse outcomes in hospitalized patients [9]. Albumin is a negative acute-phase reactant that decreases in response to inflammatory cytokines and reflects not only protein intake but also systemic inflammation, impaired hepatic synthesis, fluid redistribution, and catabolic stress [28,29]. In heart failure populations, albumin has even been reported to provide prognostic information comparable to composite malnutrition indices, suggesting that a substantial part of the prognostic signal of CONUT may be mediated by inflammation-related hypoalbuminemia combined with reduced protein reserves [30]. Total cholesterol may reflect metabolic reserve and substrate availability, and hypocholesterolemia has been associated with alterations in membrane integrity, immune responses, and advanced frailty states [31]. However, cholesterol concentrations are also susceptible to changes induced by lipid-lowering therapy or acute inflammatory responses, which may explain why indices that do not include cholesterol, such as the Geriatric Nutritional Risk Index (GNRI), sometimes achieve better risk reclassification despite similar discrimination [32]. Finally, total lymphocyte count reflects immune competence and frequently decreases during systemic inflammation, severe infection, and catabolic stress. Lymphopenia has been associated with increased mortality in sepsis and other acute conditions, supporting its role as a marker of immune dysfunction [33]. Consistent with the inflammation–malnutrition framework, studies in diabetic patients undergoing coronary angiography have shown a synergistic association between CONUT-defined malnutrition and elevated hs-CRP levels with increased mortality, suggesting that inflammation may amplify the prognostic impact of nutritional impairment [16]. Similar mediation by inflammatory indices has also been suggested in cardiovascular populations [25] and COVID-19 cohorts [34]. Taken together, these observations support interpreting CONUT as an integrated marker of immuno-metabolic vulnerability, particularly informative in hospitalized populations where multiple sources of inflammation and catabolic stress coexist.

In line with these mechanistic considerations, the observed association between CONUT and prolonged hospital stay can also be interpreted within this integrated pathophysiological framework. Specifically, the association between CONUT and prolonged hospital stay observed in the present study is also biologically and clinically plausible. LOS functions as a practical proxy for complication burden, functional decline, and healthcare resource utilization, and poor nutritional status and systemic illness severity have long been recognized as determinants of these outcomes [35]. Mechanistically, prolonged hospitalization in patients with higher CONUT scores may reflect downstream complications such as nosocomial infections, impaired wound healing, pressure injuries, or delayed functional recovery [36,37]. Evidence supporting this pathway has been reported in several clinical contexts. In patients with first-ever ischemic stroke, higher admission CONUT scores have been associated not only with mortality but also with increased rates of in-hospital complications, including pneumonia, urinary tract infection, and pressure ulcers [38]. Similarly, in COVID-19 cohorts, CONUT and related laboratory parameters have demonstrated concurrent predictive value for thrombotic complications and mortality alongside inflammatory biomarkers, supporting a pathophysiological framework in which immune dysfunction, endothelial injury, and reduced physiological reserve converge to influence clinical severity and resource utilization [18,19,34].

Beyond these mechanistic insights, positioning CONUT within the landscape of existing nutritional assessment tools is essential to better define its clinical value in routine practice [39]. Comparative analyses against established tools provide key insights into its utility. Traditional multidimensional instruments for nutritional screening (NRS-2002, MUST, MST, and MNA-SF) or assessment (SGA and GLIM) rely on clinical interviews and subjective parameters (e.g., recent weight loss or dietary intake). Although these approaches are specific for phenotypic malnutrition, they are time-consuming, subject to inter-observer variability, and potentially confounded by clinical conditions such as fluid overload in heart failure [10,22,30]. In contrast, CONUT provides a fully automated, laboratory-based and objective complementary approach that enables rapid and reproducible risk stratification. For example, in patients with chronic heart failure, CONUT showed the highest prognostic accuracy for mortality when compared with multiple biochemical indices (GNRI and PNI), MUST, and SGA [30]. Similarly, in hospitalized populations, CONUT has outperformed traditional assessment (SGA and NSA) tools in ROC analyses for mortality prediction [22]. Notably, CONUT tends to classify a higher proportion of patients as being at moderate-to-severe nutritional risk compared to subjective tools such as SGA [22], with a similar trend in nutritional screening (NRS-2002 and MUST) tools in other works [10,30]. However, when evaluated strictly as a diagnostic tool for malnutrition, CONUT shows limited validity compared with established frameworks. Studies using GLIM as the reference standard consistently report low concordance, poor sensitivity, and lack of independent association, with very low agreement and reduced sensitivity for detecting true malnutrition [40,41,42]. In contrast, indices such as GNRI and, to a lesser extent, PNI, demonstrate better diagnostic performance and stronger association with GLIM-defined malnutrition [42]. Similarly, comparisons with clinical assessment tools such as SGA show that CONUT has inferior validity for accurately characterizing nutritional status, despite detecting a higher proportion of patients at risk [10,22,30]. This discrepancy reflects a key conceptual difference: while GLIM and SGA capture nutritional status through phenotypic and etiological criteria, CONUT is driven by biochemical parameters influenced by inflammation and disease severity, limiting its diagnostic accuracy. Therefore, CONUT should not be considered a diagnostic tool, but rather a prognostic biomarker, whereas GLIM and SGA remain the reference standards for nutritional assessment.

Taken together, these findings support the prognostic robustness of CONUT and highlight its potential for practical implementation within routine hospital care. Because it can be automatically calculated from routine laboratory tests obtained at admission, CONUT provides a scalable and cost-effective model for population-level clinical risk surveillance that does not depend on nursing workload or subjective bedside assessments. It should be noted that some patients with long-term chronic malnutrition may not be fully identified by this tool, as certain biochemical parameters may stabilize over time [43]. However, this limitation reinforces the concept that CONUT should not be interpreted as a diagnostic tool for malnutrition, but rather as a marker of clinical vulnerability.

The independent association with mortality and hospital length of stay, together with the modest but statistically significant improvement in model discrimination after incorporating CONUT, supports its role as a complementary prognostic instrument within hospital triage and inpatient workflows. However, the magnitude of this improvement was modest, and such changes in AUC may have limited clinical impact on individual decision-making, particularly when baseline models already show moderate discrimination. In this context, the added value of CONUT may lie in enhancing overall risk stratification and enabling early identification of vulnerable patients, rather than acting as a standalone determinant for clinical decisions.

Moreover, the observation that only a minority of moderate-to-severe risk patients were referred to the Nutrition Unit or received nutritional support suggests a substantial implementation gap between risk identification and clinical intervention. Similar findings have been reported in Spanish hospital settings, where automated tools have demonstrated operational advantages over more labor-intensive nutritional assessments while maintaining competitive prognostic performance [22]. In this context, CONUT could serve as an efficient trigger for early multidisciplinary assessment and preventive strategies aimed at reducing complications and improving outcomes in vulnerable hospitalized patients.

## 5. Strengths, Limitations, and Future Directions

This study has several strengths. First, it includes a relatively large cohort of hospitalized patients (n = 671) derived from routine clinical practice in a tertiary university hospital, with representation from multiple specialties including internal medicine, gastroenterology, general surgery, pulmonology, cardiology, and oncology. This heterogeneity increases the clinical relevance of the findings and reflects real-world inpatient case-mix. Second, the analytical strategy incorporated hierarchical multivariable models and evaluated CONUT both as a continuous variable and as categorical severity groups, allowing complementary interpretations of its prognostic value. In addition, the assessment of model discrimination using ROC curves and statistical comparison of AUCs through the DeLong test strengthens the methodological robustness and interpretability of the predictive analyses.

However, several limitations must be acknowledged. A primary limitation is the retrospective nature of this study, which precluded the assessment of body composition. Because data were obtained from electronic health records, objective morphofunctional measurements (such as bioelectrical impedance analysis, muscle ultrasound, or handgrip strength) were not available for this cohort. Consequently, it was not possible to fully disentangle true sarcopenia or pure energy–protein deficit from biochemical alterations primarily driven by the acute inflammatory response. Another important limitation is the restriction of the analysis to patients with CONUT ≥ 6. This threshold, determined by the configuration of the laboratory information system, limited the evaluation to a preselected higher-risk population, thereby reducing generalizability and preventing assessment across the full spectrum of nutritional risk. As a result, potential non-linear associations and threshold effects could not be explored, in contrast to broader multicenter registries [10,23]. In addition, the single-center design may limit external validity in hospitals with different patient profiles or clinical management protocols. Finally, as an observational study, this analysis cannot establish causal relationships or determine whether CONUT-guided interventions directly improve outcomes.

Future research should therefore focus on prospective, multicenter cohorts that include unselected hospitalized populations across the entire spectrum of CONUT values. This would allow for the refinement of risk thresholds and an improvement in external validity. Comparative analyses with traditional screening tools (e.g., NRS-2002 or MUST) and other biochemical indices (e.g., GNRI) are also warranted to clarify their relative prognostic performances. Crucially, given the complex interplay between malnutrition and systemic inflammation, future predictive models and clinical trials should aim to integrate automated biochemical screening tools like CONUT with bedside morphofunctional assessments. This combined approach, aligning with the GLIM diagnostic framework, would help identify which patients are most likely to benefit from specific nutritional therapies [16,25]. Ultimately, prospective interventional studies are needed to assess whether a CONUT-guided care pathway, when complemented by a comprehensive etiologic and phenotypic evaluation, can effectively reduce mortality, complications, and hospital length of stay.

## 6. Conclusions

**Figure 4:**
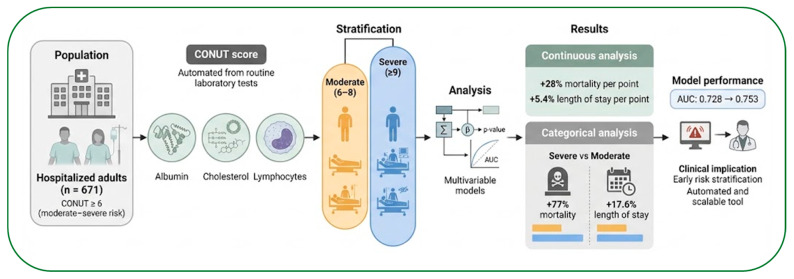
Graphical summary of the prognostic value of the CONUT score in hospitalized patients.

In this real-world cohort of hospitalized adults, the CONUT score was independently associated with in-hospital mortality and prolonged length of stay, supporting its role as a robust prognostic marker (Figure 4). Although the improvement in model discrimination was modest, its statistical significance reinforces the incremental value of CONUT beyond routinely available clinical variables. These findings support the concept that CONUT reflects a state of immuno-metabolic vulnerability driven by the interplay between nutritional depletion and systemic inflammation.

From a clinical perspective, the automated calculation of CONUT from routine admission laboratory tests enables a scalable and cost-effective strategy for early risk stratification. Rather than serving as a standalone trigger for nutritional intervention, a high CONUT score should be interpreted as a pragmatic warning signal that prompts comprehensive, individualized assessment, ideally including objective evaluation of body composition and muscle mass. In this context, CONUT may facilitate the early identification of vulnerable patients and support more targeted, multidisciplinary clinical decision-making in routine hospital care.

## Figures and Tables

**Table 1:** Baseline characteristics and clinical outcomes according to CONUT nutritional status.

Variable	Overall	CONUT Moderate (6–8)	CONUT Severe (9–10)
**Population Characteristics**			
N	671	497	174
Age, mean (SD)	74.2 (14.4)	75.0 (14.3) ^a^	72.0 (14.7) ^b^
Women sex, n (%)	274 (40.7)	218 (32.3) ^a^	56 (8.3) ^b^
**Hospital admission**			
Medical admission, n (%)	544 (80.7)	404 (59.9)	140 (20.8)
Referral to Nutrition Unit, n (%)	102 (15.1)	69 (10.2)	33 (4.9)
Received nutritional support, n (%)	123 (18.3)	86 (12.8)	37 (5.5)
Surgical admission, n (%)	130 (19.3)	94 (13.9)	36 (5.4)
Referral to Nutrition Unit, n (%)	34 (5.1)	20 (3.0)	14 (2.1)
Received nutritional support, n (%)	39 (5.8)	24 (3.6)	15 (2.2)
**ICD-10-based diagnostic**			
Diseases of the respiratory system, n (%)	171 (25.5)	129 (19.1)	42 (6.2)
Diseases of the digestive system, n (%)	168 (25.0)	114 (16.9)	54 (8.0)
Diseases of the circulatory system, n (%)	104 (15.5)	86 (12.8)	18 (2.7)
Neoplasms, n (%)	79 (11.8)	55 (8.2)	24 (3.6)
Diseases of the genitourinary system, n (%)	67 (10.0)	56 (8.3)	11 (1.6)
Certain infectious and parasitic diseases, n (%)	30 (4.5)	15 (2.2)	15 (2.2)
Blood, hematopoietic, and immune system disorders, n (%)	10 (1.5)	9 (1.3)	1 (0.1)
Other chapters, n (%)	45 (6.7)	34 (5.0)	11 (1.6)
**Hospital Admission Service**			
Internal Medicine, n (%)	261 (38.7)	199 (29.5)	62 (9.2)
Gastroenterology, n (%)	99 (14.7)	64 (9.5)	35 (5.2)
General Surgery, n (%)	74 (11.0)	52 (7.7)	22 (3.3)
Pulmonology, n (%)	46 (6.8)	36 (5.3)	10 (1.5)
Cardiology, n (%)	35 (5.2)	31 (4.6)	4 (0.6)
Medical Oncology, n (%)	33 (4.9)	23 (3.4)	10 (1.5)
Infectious Diseases, n (%)	26 (3.9)	19 (2.8)	7 (1.0)
Nephrology, n (%)	21 (3.1)	18 (2.7)	3 (0.4)
Hematology, n (%)	20 (3.0)	11 (1.6)	9 (1.3)
Urology, n (%)	19 (2.8)	15 (2.2)	4 (0.6)
Angiology and Vascular Surgery, n (%)	12 (1.8)	5 (0.7)	7 (1.0)
Other Surgical Specialties, n (%)	18 (2.7)	16 (2.4)	2 (0.3)
Other Medical Specialties, n (%)	10 (1.5)	9 (1.3)	1 (0.1)
**Clinical Outcomes**			
Length of stay, days (median [IQR])	11 (12.0)	11.0 (12.0) ^a^	13.0 (14.3) ^a^
In-hospital mortality, n (%)	132 (19.6)	85 (12.6) ^a^	47 (7.0) ^b^

Different letters indicate statistically significant differences between groups at *p* < 0.05. Data are presented as n (%) unless otherwise indicated. Age is shown as mean (SD) and length of stay as median (IQR). SD: standard deviation; IQR: interquartile range.

**Table 2:** Hierarchical Multivariable Logistic Regression Models for In-Hospital Mortality.

Variable	Parameter	Model 1	Model 2	Model 3
**Age (per year)**	**OR (95% CI)**	1.02 (1.01–1.04)	1.03 (1.01–1.05)	1.03 (1.01–1.05)
	***p*** **-value**	0.008	<0.001	<0.001
**Biological sex**	**OR (95% CI)**	0.87 (0.56–1.31)	1.16 (0.75–1.79)	1.21 (0.79–1.86)
	***p*** **-value**	0.511	0.502	0.372
**Admission service**	**OR (95% CI)**	Global	Global	Global
	***p*** **-value**	<0.001	<0.001	<0.001
**ICD-10-based diagnostic**	**OR (95% CI)**	Global	Global	Global
	***p*** **-value**	0.335	0.343	0.353
**CONUT (per point)**	**OR (95% CI)**	—	1.28 (1.13–1.46)	—
	***p*** **-value**	—	<0.001	—
**CONUT (categorical)**	**OR (95% CI)**	—	—	1.77 (1.12–2.81)
	***p*** **-value**	—	—	0.004

Values are presented as odds ratios (OR) with 95% confidence intervals (CI) obtained from hierarchical multivariable logistic regression models for in-hospital mortality (deceased vs. alive). Model 1 included clinical variables (age, sex, ICD-10–based diagnosis, and admission service). Model 2 consisted of Model 1 plus CONUT as a continuous variable (per-point increase). Model 3 consisted of Model 1 plus CONUT as a categorical variable (severe vs. moderate). Reference categories were female sex, internal medicine admission, respiratory diseases (ICD-10 chapter), and moderate CONUT score.

**Table 3:** Multivariable Linear Regression Models Assessing the Association Between CONUT Score and Log-Transformed Length of Hospital Stay.

Variable		Model A	Model B	Model C
**Age (per year)**	**β**	−0.00585 (−0.0106 to −0.00109)	−0.00584 (−0.0106 to −0.00111)	−0.00559 (−0.0103 to −0.00084)
	**% Change**	−0.6% (−1.1 to −0.1)	−0.6% (−1.1 to −0.1)	−0.6% (−1.0 to −0.1)
	***p***	0.016	0.016	0.021
**Biological sex**	**β**	0.10955 (−0.0164 to 0.23551)	0.0843 (−0.0425 to 0.2112)	0.0916 (−0.0350 to 0.2181)
	**% Change**	11.6% (−1.6 to 26.6)	+8.8% (−4.2 to 23.5)	+9.6% (−3.4 to 24.4)
	***p***	0.088	0.192	0.156
**Admission service**	**β**	Global effect	Global effect	Global effect
	**% Change**	Global effect	Global effect	Global effect
	***p***	0.002	0.002	0.101
**ICD-10-based diagnostic**	**β**	Global effect	Global effect	Global effect
	**% Change**	Global effect	Global effect	Global effect
	***p***	0.130	0.094	0.001
**CONUT (per point)**	**β**	—	0.05227 (0.0130 to 0.0915)	—
	**% Change**	—	+5.4% (1.3 to 9.6)	—
	***p***	—	0.009	—
**CONUT (categorical)**	**β**	—	—	0.16197 (0.0187 to 0.3052)
	**% Change**	—	—	+17.6% (1.9 to 35.7)
	***p***	—	—	0.027

Values are β coefficients from multivariable linear regression models with log-transformed length of hospital stay as the dependent variable. Results are expressed as β (95% CI) and percentage change in LOS, calculated as (e^β − 1) × 100. All models were adjusted for age, sex (reference: female), ICD-10-based diagnostic (reference: respiratory diseases), and admission service (reference: internal medicine). Model A included clinical variables (age, sex, ICD-10–based diagnosis, and admission service). Model B consisted of Model A plus CONUT as a continuous variable (per-point increase). Model C consisted of Model A plus CONUT as a categorical variable (severe vs. moderate). Reference categories were female sex, internal medicine admission, respiratory diseases (ICD-10 chapter), and moderate CONUT score. Model assumptions were verified through residual diagnostics and variance inflation factors.

## Data Availability

The original contributions presented in the study are included in the article; further inquiries can be directed to the corresponding authors.

## Supplementary Materials

The following supporting information can be downloaded at: https://www.mdpi.com/article/10.3390/nu18081249/s1, Table S1. Association of diagnostic categories and admission service with in-hospital mortality. Table S2. Association of diagnostic categories and admission service with Length of Hospitality Stay.
