# Supplementary material for: Incremental Prognostic Value of the CONUT Score for In-Hospital Mortality and Length of Stay in Hospitalized Patients
Source: Nutrients. 2026 Apr 15;18(8):1249. doi: 10.3390/nu18081249 (PMC13118508; doi:10.3390/nu18081249)
Supplement: Supplementary file 1 [file nutrients-18-01249-s001.zip › nutrients-4239655-supplementary.pdf]

Table S1. Association of diagnostic categories and admission service with in-hospital mortality.

Variable	Parameter	Model 1	Model 2	Model 3
**ICD-based diagnostic**				
Certain Infectious and Parasitic Diseases	**OR (95% CI)**	1.8509 (0.69047–4.9618)	1.56379 (0.5737–4.2625)	1.6032 (0.59043–4.353)
Diseases of the Circulatory System	**OR (95% CI)**	**2.1807 (1.05183–4.5209)***	**2.49819 (1.1782–5.2970)***	**2.3361 (1.11638–4.888)***
Diseases of the Digestive System	**OR (95% CI)**	1.6537 (0.70259–3.8923)	1.67803 (0.7022–4.0100)	1.6966 (0.71616–4.019)
Diseases of the Genitourinary System	**OR (95% CI)**	1.4120 (0.63559–3.1369)	1.60284 (0.7145–3.5958)	1.4886 (0.66812–3.317)
Blood, hematopoietic, and immune system disorders	**OR (95% CI)**	0.4487 (0.04989–4.0347)	0.58829 (0.0669–5.1764)	0.5393 (0.06032–4.822)
Neoplasms	**OR (95% CI)**	1.4095 (0.54659–3.6348)	1.38631 (0.5252–3.6593)	1.3667 (0.52417–3.564)
Other Chapters	**OR (95% CI)**	0.7793 (0.26016–2.3344)	0.89985 (0.3009–2.6914)	0.8247 (0.27776–2.449)
**Admission Service**				
Angiology and Vascular Surgery	**OR (95% CI)**	1.5474 (0.39789–6.0179)	1.18953 (0.2937–4.8175)	1.1922 (0.29452–4.826)
Cardiology	**OR (95% CI)**	**0.2156 (0.05727–0.8120)***	**0.22661 (0.0593–0.8655)***	**0.2201 (0.05813–0.833)***
Gastroenterology	**OR (95% CI)**	1.0567 (0.44385–2.5156)	0.99538 (0.4117–2.4068)	0.9888 (0.41158–2.376)
General Surgery	**OR (95% CI)**	**0.1812 (0.04835–0.6787)***	**0.16744 (0.0442–0.6346)***	**0.1760 (0.04672–0.663)***
Hematology	**OR (95% CI)**	**4.9189 (1.47196–16.4379)***	**4.75258 (1.3968–16.1707)***	**4.5072 (1.33956–15.165)***
Infectious Diseases	**OR (95% CI)**	0.9468 (0.29527–3.0362)	0.76554 (0.2257–2.5964)	0.8785 (0.26950–2.864)
Medical Oncology	**OR (95% CI)**	**7.7845 (2.95059–20.5377)***	**7.94233 (2.9506–21.3792)***	**7.8642 (2.94540–20.998)***
Nephrology	**OR (95% CI)**	0.2087 (0.02597–1.6768)	0.19407 (0.0239–1.5774)	0.2152 (0.02677–1.730)
Other Medical Specialties	**OR (95% CI)**	3.0025 (0.62072–14.5230)	2.99901 (0.6018–14.9450)	3.2093 (0.65050–15.834)
Other Surgical Specialties	**OR (95% CI)**	1.0472 (0.26911–4.0748)	1.18656 (0.3016–4.6681)	1.1232 (0.28755–4.387)
Pulmonology	**OR (95% CI)**	0.4695 (0.15422–1.4293)	0.49801 (0.1619–1.5318)	0.4936 (0.16154–1.508)
Urology	**OR (95% CI)**	0.5982 (0.12714–2.8144)	0.56345 (0.1187–2.6747)	0.6158 (0.13122–2.890)

Values are presented as odds ratios (OR) with 95% confidence intervals (CI) obtained from hierarchical multivariable logistic regression models for in-hospital mortality (deceased vs. alive). Model 1 included clinical variables (age, sex, ICD-10–based diagnosis, and admission service). Model 2 consisted of Model 1 plus CONUT as a continuous variable (per-point increase). Model 3 consisted of Model 1 plus CONUT as a categorical variable (severe vs. moderate). Reference categories were female sex, internal medicine admission, respiratory diseases (ICD-10 chapter), and moderate malnutrition. * Indicates statistical significance ($p < 0.05$).

Table S2. Association of diagnostic categories and admission service with Length of Hospitality Stay.

Variable	Parameter	Model 1	Model 2	Model 3
**ICD-based diagnostics**				
Certain Infectious and Parasitic Diseases	β	0.12151 (-0.1985 – 0.44148)	0.07893 (-0.2412 – 0.39908)	0.08039 (-0.2407 – 0.4015)
	**% Change**	12.92 (-18.00 – 55.50)	8.20 (-21.43 – 49.02)	8.37 (-21.41 – 49.39)
Diseases of the Circulatory System	β	**0.26048 (0.0244 – 0.49654)***	**0.28068 (0.0452 – 0.51617)***	**0.27494 (0.0392 – 0.5106)***
	**% Change**	**29.75 (2.47 – 64.32)***	**32.40 (4.63 – 67.54)***	**31.63 (4.00 – 66.61)***
Diseases of the Digestive System	β	**0.35691 (0.0962 – 0.61757)***	**0.35296 (0.0934 – 0.61247)***	**0.36024 (0.1003 – 0.6201)***
	**% Change**	**42.86 (10.10 – 85.45)***	**42.30 (9.79 – 84.52)***	**43.39 (10.55 – 85.92)***
Diseases of the Genitourinary System	β	0.09600 (-0.1537 – 0.34571)	0.11678 (-0.1323 – 0.36587)	0.10900 (-0.1402 – 0.3582)
	**% Change**	10.08 (-14.25 – 41.27)	12.39 (-12.39 – 44.20)	11.52 (-13.08 – 43.07)
Blood, hematopoietic, and immune system disorders	β	0.24187 (-0.2750 – 0.75874)	0.27275 (-0.2423 – 0.78783)	0.27298 (-0.2431 – 0.7890)
	**% Change**	27.35 (-24.07 – 113.54)	31.36 (-21.52 – 119.84)	31.40 (-21.57 – 120.13)
Neoplasms	β	0.21503 (-0.0686 – 0.49868)	0.22551 (-0.0570 – 0.50801)	0.22026 (-0.0626 – 0.5031)
	**% Change**	23.98 (-6.63 – 64.63)	25.30 (-5.54 – 66.17)	24.64 (-6.06 – 65.41)
Other Chapters	β	**0.34217 (0.0398 – 0.64458)***	**0.36462 (0.0631 – 0.66614)***	**0.34659 (0.0451 – 0.6481)***
	**% Change**	**40.81 (4.06 – 90.52)***	**44.01 (6.52 – 94.68)***	**41.42 (4.61 – 91.20)***
**Admission Service**				
Angiology and Vascular Surgery	β	0.16350 (-0.6493 – 0.32232)	0.20826 (-0.6931 – 0.27655)	-0.22587 (-0.7134 – 0.2616)
	**% Change**	17.77 (-47.77 – 38.05)	23.15 (-49.99 – 31.85)	-20.22 (-51.04 – 29.88)
Cardiology	β	-0.29928 (-0.6301 – 0.03157)	-0.28214 (-0.6118 – 0.04748)	-0.28979 (-0.6198 – 0.0402)
	**% Change**	-25.85 (-46.71 – 3.21)	-24.56 (-45.75 – 4.86)	-25.15 (-46.10 – 4.10)
Gastroenterology	β	**-0.47885 (-0.7511 – -0.20658)***	**-0.48735 (-0.7585 – -0.21622)***	**-0.49361 (-0.7654 – -0.2218)***
	**% Change**	**-38.04 (-52.79 – -18.67)***	**-38.54 (-53.15 – -19.45)***	**-38.98 (-53.51 – -19.88)***
General Surgery	β	0.12770 (-0.4005 – 0.14510)	0.14059 (-0.4123 – 0.13117)	-0.13463 (-0.4067 – 0.1374)
	**% Change**	13.62 (-32.96 – 15.61)	15.10 (-33.76 – 14.02)	-12.61 (-33.44 – 14.73)
Hematology	β	**0.51836 (0.0977 – 0.93902)***	**0.49503 (0.0759 – 0.91417)***	**0.48350 (0.0630 – 0.9040)***
	**% Change**	**67.93 (10.27 – 156.87)***	**64.03 (7.88 – 149.44)***	**62.17 (6.50 – 146.94)***
Infectious Diseases	β	0.02796 (-0.3647 – 0.30883)	0.03957 (-0.3750 – 0.29582)	-0.03089 (-0.3667 – 0.3049)
	**% Change**	2.84 (-30.55 – 36.17)	4.03 (-31.30 – 34.40)	-3.04 (-30.72 – 35.64)
Medical Oncology	β	0.14702 (-0.1897 – 0.48377)	0.13191 (-0.2035 – 0.46735)	0.13810 (-0.1977 – 0.4739)
	**% Change**	15.84 (-17.29 – 62.22)	14.10 (-18.44 – 59.54)	14.80 (-17.92 – 60.60)
Nephrology	β	0.13040 (-0.2510 – 0.51182)	0.12643 (-0.2533 – 0.50616)	0.14115 (-0.2392 – 0.5215)
	**% Change**	13.93 (-22.18 – 66.82)	13.48 (-22.36 – 65.86)	15.16 (-21.28 – 68.43)

Other Medical Specialties	**β**	0.25123 (-0.2881 – 0.79059)	0.25378 (-0.2832 – 0.79074)	0.27693 (-0.2613 – 0.8151)
	**% Change**	28.57 (-25.01 – 120.54)	28.89 (-24.68 – 120.70)	31.91 (-22.96 – 126.01)
Other Surgical Specialties	**β**	0.24548 (-0.1550 – 0.64597)	0.27023 (-0.1289 – 0.66937)	0.26917 (-0.1307 – 0.6690)
	**% Change**	27.81 (-14.35 – 90.80)	31.02 (-12.09 – 95.29)	30.87 (-12.25 – 95.24)
Pulmonology	**β**	0.13299 (-0.1431 – 0.38966)	0.13279 (-0.1325 – 0.39806)	0.13343 (-0.1323 – 0.3991)
	**% Change**	14.22 (-13.33 – 47.64)	14.20 (-12.42 – 48.90)	14.28 (-12.39 – 49.03)
Urology	**β**	0.08532 (-0.2995 – 0.47018)	0.08606 (-0.2971 – 0.46920)	0.09198 (-0.2918 – 0.4757)
	**% Change**	8.91 (-25.88 – 60.03)	8.99 (-25.71 – 59.89)	9.63 (-25.33 – 60.95)

Values are β coefficients from multivariable linear regression models with log-transformed length of hospital stay as the dependent variable. Results are expressed as β (95% CI) and percentage change in LOS, calculated as $(e^{\beta} - 1) \times 100$. All models were adjusted for age, sex (reference: female), ICD-10-based diagnostic (reference: respiratory diseases), and admission service (reference: internal medicine). Model A included clinical variables (age, sex, ICD-10–based diagnosis, and admission service). Model B consisted of Model A plus CONUT as a continuous variable (per-point increase). Model C consisted of Model A plus CONUT as a categorical variable (severe vs. moderate). Reference categories were female sex, internal medicine admission, respiratory diseases (ICD-10 chapter), and moderate malnutrition. Model assumptions were verified through residual diagnostics and variance inflation factors. * Indicates statistical significance ($p < 0.05$).
